# Cofactor Engineering Strategy of Food-Grade Microorganisms: Redox Homeostasis Regulation and Functional Components Biofortification

**DOI:** 10.3390/microorganisms14050992

**Published:** 2026-04-28

**Authors:** Di Zhang, Haoran Wang, Xin Song, Yongjun Xia, Guangqiang Wang, Zhiqiang Xiong, Lianzhong Ai

**Affiliations:** 1 Shanghai Engineering Research Center of Food Microbiology, School of Health Science and Engineering, University of Shanghai for Science and Technology, Shanghai 200093, China; dz_edu@163.com (D.Z.);; 2 School of Agriculture and Biology, Shanghai Jiao Tong University, Shanghai 200240, China

**Keywords:** cofactor engineering strategy, food-grade microorganisms, redox homeostasis, metabolic engineering, functional ingredients

## Abstract

Food-grade microorganisms utilize core cofactors, such as nicotinamide adenine dinucleotide (NAD) and its phosphate form (NADP), to mediate redox reactions and regulate energy metabolism homeostasis as well as biosynthesis of functional components. In metabolic engineering, perturbation of the NAD(P)^+^/NAD(P)H network may significantly disrupt intracellular redox homeostasis, leading to impaired strain growth and limited synthesis of targeted functional products. This review systematically examines the latest research progress in the field of food-grade microbial cofactor engineering, focusing on the key mechanisms and synergistic pathways of core strategies, such as metabolic flux optimization and cofactor regeneration systems, in maintaining cellular redox homeostasis and enhancing the biosynthesis of functional ingredients. Future research should focus on exploring the potential for integrating multi-omics approaches and intelligent control technologies, proposing innovative approaches to address the challenges of industrialized production, and providing theoretical support for food biomanufacturing.

## 1. Introduction

Cofactors, such as metal ions and organic compounds, are vital for the catalytic systems of food-grade microorganisms. They assist enzymes in redox reactions and metabolic processes, ensuring the proper functioning of microbial metabolic networks. Among these, nicotinamide adenine dinucleotide (NAD) and its phosphorylated form (NADP) are core redox cofactors that play a vital role in energy metabolism and the biosynthesis of functional components. NAD and NADP participate in biosynthesis by undergoing reversible conversion between their oxidized (NAD+/NADP+) and reduced (NADH/NADPH), which is the basis for maintaining the intracellular redox balance of food-grade microorganisms [1]. Although NAD and NADP share the same nicotinamide-adenine backbone structure, the phosphate group at the 2′-position of the NADP molecule imparts a unique metabolic partitioning function [2]. Specifically, NADH, which is mainly generated in the glycolysis pathway and the tricarboxylic acid (TCA) cycle, is mainly responsible for driving catabolic reactions, providing energy for microbial growth and reproduction by participating in oxidative phosphorylation; produced by glycolysis and the tricarboxylic acid (TCA) cycle, is mainly responsible for driving catabolic reactions, providing energy for microbial growth and reproduction by participating in oxidative phosphorylation; while NADPH, which is mainly produced by the pentose phosphate pathway (PPP), is the main reducing power source for anabolic reactions, and is essential for the synthesis of high-value functional components in food fermentation, such as flavor substances, vitamins and antioxidants [3]. This metabolic compartmentalization mechanism based on NAD(P) ensures the precise distribution of carbon flux and electron flux in the food fermentation system, making the microbial cell realize the efficient coupling of energy metabolism and material synthesis [4].

In the field of food microbial metabolic engineering, the targeted modification of metabolic pathways is often used to redirect carbon flux to the synthetic pathway of target functional components, but this operation is likely to cause the imbalance of the intracellular NAD(P)^+^/NAD(P)H redox network, which further leads to a series of problems such as inhibited microbial growth, reduced strain activity, and limited synthesis of target products. For instance, in the dairy fermentation strain *Lactococcus lactis*, in order to increase the yield of the flavor substance diacetyl, the lactate dehydrogenase (LDH) gene is often knocked out to block the lactic acid synthesis pathway and redirect the carbon flux to the diacetyl synthesis branch [5]. However, the knockout of LDH will lead to the excessive accumulation of NADH in the glycolysis pathway, destroy the intracellular redox balance, and ultimately result in a significant reduction in strain growth rate and diacetyl yield. Similarly, in the process of enhancing the synthesis of functional alcohols such as 2,3-butanediol by food-grade microorganisms, a large amount of NAD(P)H is consumed, which will also break the original redox balance of the cell, cause the disorder of metabolic flow, and even lead to the accumulation of toxic by-products. Moreover, low affinity of key enzymes for cofactors in the target product synthesis pathway can result in inefficient cofactor consumption, diverting carbon flux to competitive pathways and reducing the synthesis efficiency of functional components [6].

**Figure 1:**
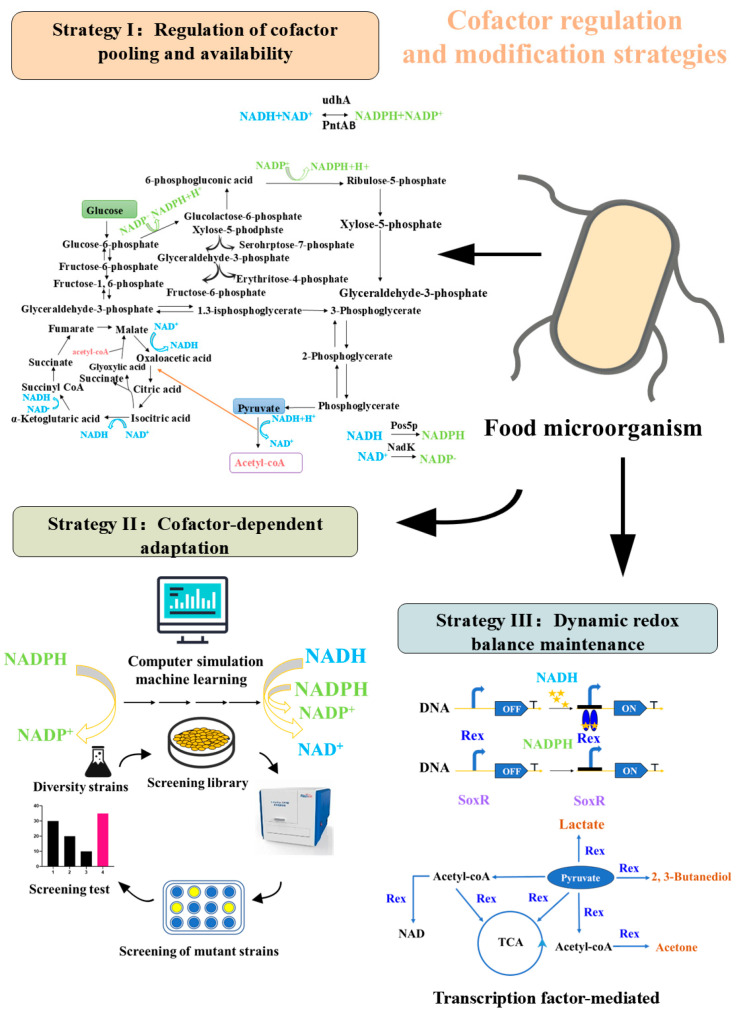
Strategies and applications of cofactor engineering. The three major strategies related to cofactors synergistically enhance functional component synthesis for food biomanufacturing. Strategy I (Regulation of cofactor pool) optimizes metabolic flux by knocking out competitive pathways (*ldh*, *pta*) and enhances NADPH supply via PPP upregulation or PNT-mediated interconversion. Strategy II (Cofactor-dependent molecular remodeling) improves enzyme efficiency through rational design of cofactor-binding sites or the use of artificial cofactor analogs. Strategy III (Dynamic redox balance) employs biosensors (Yap1p, SoxR) and transcription regulators (Rex) to achieve real-time NAD(P)H homeostasis. These strategies collectively address redox bottlenecks in the biosynthesis of functional food ingredients.

To solve the above problems caused by redox imbalance, cofactor engineering has become a key technical means in the metabolic engineering of food-grade microorganisms. Cofactor engineering optimizes intracellular cofactor levels and enzyme specificity through targeted genetic modifications, enzyme remodeling, and synthetic circuits. This restores redox homeostasis in microbial cells, improving fermentation efficiency and the synthesis of functional components. At present, three core strategies of cofactor engineering for food-grade microorganisms have been formed and applied in practice: the first is the regulation of cofactor pool and availability, which optimizes the cofactor supply by knocking out non-essential metabolic pathways, enhancing cofactor regeneration systems and mediating cofactor interconversion; the second is the cofactor-dependent enzyme molecular remodeling, which improves the matching degree between enzymes and cofactors by modifying the key enzymes in the synthetic pathway or developing artificial cofactor analogs; the third is the maintenance of dynamic redox balance, which constructs adaptive regulatory circuits based on redox-responsive components to realize the real-time regulation of NAD(P)H homeostasis during fermentation. These three strategies complement each other and form a systematic solution to overcome the redox bottleneck in food biomanufacturing, providing a new technical path for the efficient synthesis of functional components in food fermentation (Figure 1).

(1) **Regulation of cofactor pooling and availability (Strategy I):** Optimizing the biosynthetic efficiency of high-value food functional ingredients involves knocking out non-essential metabolic pathways and enhancing the pentose phosphate pathway (PPP) to increase NADPH supply. Additionally, pyridine nucleotide transhydrogenase (PNT) can be utilized to mediate the interconversion of NAD(P)H.
(2) **Cofactor-dependent changes in enzymes (Strategy II):** Rational design of key enzymes in the synthetic pathway or the development of food-compatible cofactor analogs to precisely match the biosynthetic requirements of the target product.
(3) **Dynamic redox balance maintenance (Strategy III):** Based on redox-responsive components, adaptive regulatory circuits are constructed for food microorganisms to achieve real-time maintenance of NAD(P)H homeostasis during fermentation, thus ensuring efficient synthesis of key substances.

Collectively, these three strategies provide a systematic solution to overcome the redox bottleneck in food biomanufacturing.

## 2. Targeted Regulation of the Cofactor Pool

Targeted regulation of the cofactor pool is a fundamental strategy in food-grade microbial cofactor engineering. It focuses on increasing cellular levels of essential cofactor forms (NADH or NADPH) and optimizing metabolic flux to effectively meet the cofactor demands for synthesizing target functional components (Table 1).

### 2.1. Competitive Pathways for Cofactor Knockout

Targeted knockout of key genes in non-essential metabolic pathways can effectively reduce ineffective consumption of cofactors, and thus optimize the biosynthesis of functional food components. In the practical application, the gene knockout technology for food-grade microorganisms has gradually developed from traditional homologous recombination to scarless editing and CRISPR interference (CRISPRi) technology, which not only improves the knockout efficiency, but also reduces the adverse effects of exogenous fragments on the biological safety of food-grade strains. For example, in *Lactiplantibacillus plantarum*, the knockout of the pyruvate dehydrogenase (PDH) gene can block the pyruvate oxidation pathway that consumes NAD+, reduce the competitive consumption of NAD+ in the cell, make more NAD+ participate in the synthesis of the antioxidant metabolite γ-aminobutyric acid (GABA), and thus significantly increase the yield of GABA. The expression of the phosphotransferase gene (*pta*) in the acetate synthesis pathway of *Lactobacillus acidophilus* was suppressed using CRISPRi technology, which significantly increased the availability of NADPH and promoted the synthesis of probiotic extracellular polysaccharides (EPS) [7].

LDH gene knockout has been shown to effectively redirect carbon flux in lactic acid bacteria. For instance, LDH-knockout strains of *Lactococcus lactis* exhibit a shift from homolactic to mixed-acid fermentation. Knocking out the LDH gene in lactic acid bacteria using the CRISPR/Cas9 system redirects carbon flux from lactic acid to compounds with antimicrobial activity, resulting in a 78% increase in inhibitory activity [8]. However, it is worth noting that the knockout of *ldh* gene also leads to a certain reduction in the growth rate of *Bifidobacterium*, which indicates that the knockout of competitive pathways needs to balance the severity of gene modification and the yield of target products, and avoid the excessive inhibition of microbial basic metabolism due to the knockout of genes, which affects the normal growth and reproduction of the strain. In addition, the traditional static gene knockout lacks dynamic control ability, and it is difficult to adapt to the dynamic changes in cofactor demand in different fermentation stages of industrial-scale fermentation, which limits its further application in industrial production. Therefore, the combination of dynamic regulation technology and gene knockout is the development direction of this strategy in the future.

### 2.2. Introduction of Cofactor Regeneration Reaction

The introduction of a cofactor regeneration system is a key strategy for maintaining redox homeostasis within microbial cells and enhancing the accumulation rate of high-value metabolites. By introducing exogenous enzymes to catalyze reactions or enhancing endogenous metabolic pathways, the required forms of cofactors (such as NADH or NADPH) can be efficiently regenerated. This maintains a high ratio of reduced to oxidized cofactors within the cell, thereby meeting the substantial demand for reducing power required for the synthesis of target products. For example, a NADH regeneration system in *L. plantarum* was successfully developed, by the expression of the *fdh*, which encoded formate dehydrogenase (FDH) from Candida. This system significantly increased the intracellular levels of NADH and the NADH/NAD^+^ ratio, leading to a 130.0% increase in yield and productivity of the natural preservative benzoic acid [9]. The expression of FDH was a promising strategy for NADH regeneration and the production of NADH-dependent microbial metabolites in *L. plantarum*. FDH not only utilized self-generated carbon dioxide to provide non-toxic assistance but also effectively alleviated substrate inhibition [10]. However, its effectiveness is limited to formate-rich conditions.

NADH oxidase (NOX) directly achieves efficient conversion of NADH through oxygen catalysis, thereby regulating the NAD+/NADH ratio. Overexpression of NOX promoted the shift in aerobic glucose metabolism from lactic acid fermentation to mixed acid fermentation, and the extent of this shift was positively correlated with enzyme activity [11]. Overexpression of NOX accelerated NADH oxidation, reduced the NADH/NAD+ ratio, and relieved feedback inhibition on pyruvate metabolism. This metabolic shift redirected carbon sources from lactate synthesis to EPS biosynthesis, resulting in a 46% increase in EPS production in *Lacticaseibacillus casei* compared with the wild type [12]. Although this endogenous cofactor engineering strategy reduces growth rates due to the redistribution of metabolic flux, it significantly lowers the cost of biosynthetic components compared with the exogenous addition of electron acceptors or the construction of complex co-culture systems. This approach concurrently reduces the generation of metabolic by-products.

For NADPH-dependent metabolic pathways, enhancing the carbon flux of the pentose phosphate pathway (PPP) is the commonly used strategy. PPP is the main endogenous pathway for microbial cells to produce NADPH. Key enzymes, such as glucose-6-phosphate dehydrogenase (G6PD) and 6-phosphogluconate dehydrogenase, catalyze the oxidative decarboxylation of glucose-6-phosphate and 6-phosphogluconate, respectively, leading to substantial NADPH generation. Overexpression of the key enzyme glucose-6-phosphate dehydrogenase (G6PD) in the PPP, which could significantly enhance the efficiency of antimicrobial peptide synthesis in the *Saccharomyces cerevisiae var. boulardii* [13]. These antimicrobial peptides can be directly used as natural preservatives in fermented meat products, which can replace chemical preservatives and improve the safety of food. Overexpression of malic enzyme (ME) significantly increased NADPH levels in lactic acid bacteria, markedly enhancing their antioxidant capacity. Based on the improved antioxidant capacity observed in laboratory studies, this strategy has been proposed for potential application in the production of fermented sausages to inhibit lipid peroxidation [14]. Engineering modifications to glutamate dehydrogenase (GDH) could significantly reduce the accumulation of off-flavor compounds in fermented meat products [15]. In addition to the intracellular regeneration system, the cell-free NADPH regeneration system has also attracted extensive attention. The cell-free system has the advantages of no cell growth limitation, easy regulation of reaction conditions and high catalytic efficiency. Compared with whole-cell bioconversion of target substrates, NADPH regeneration reactions offer greater flexibility and a wider range of options for cell-free systems. Alcohol dehydrogenase (ADH) from *Saccharomyces cerevisiae* was coupled with terpene synthase to construct a self-sustaining NADPH regeneration system, successfully achieving efficient synthesis of limonene [16], but scalability is challenged by enzyme costs.

**Table 1:** Application of NAD and NADP synthase in increasing product yield.

Enzymes	Host Bacteria	Cofactor	Target Products	Productivity	Reference
FDH	*Lactiplantibacillus plantarum*	NAD+/NADH	benzoic acid	Production increased by 130.0%	[9]
NOX	*Lacticaseibacillus casei*	NAD+/NADH	Exopolysaccharide	Production increased by 46.0%	[12]
	-	NAD+/NADH	Shift in fermentation	Lactic acid fermentation to mixed acid fermentation	[11]
G6PD	*Saccharomyces boulardii*	NADP+/NADPH	Antimicrobial peptides	Production increased by 28.30%	[13]
ME	-	NADP+/NADPH	-	Inhibition of lipid peroxidation in fermented sausages	[14]
GDH	-	NADP+/NADPH	-	Reducing the accumulation of off-flavors in fermented meat	[15]
ADH	*Saccharomyces cerevisiae*	NADP+/NADPH	limonene	-	[16]

### 2.3. PNT Mediates the Conversion of NAD to NADP

**Figure 2:**
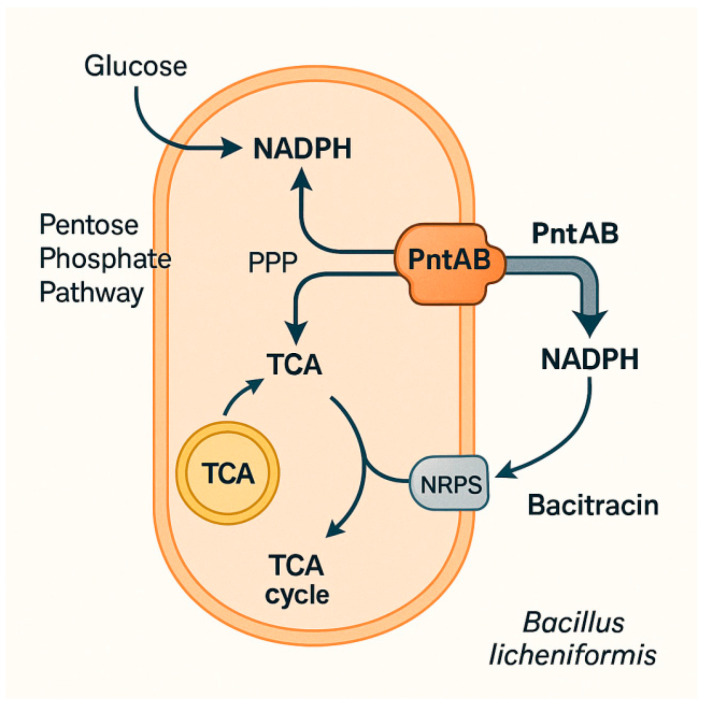
Pyridine nucleotide transhydrogenase (PntAB) mediates the interconversion of NADH and NADP^+^ to regulate bacitracin production. PntAB catalyzes the energy-dependent transfer of reducing equivalents from NADH to NADP^+^, generating NADPH to support the biosynthesis of NADPH-dependent secondary metabolites.

In addition to optimizing metabolic flux to enhance the synthesis of a single cofactor form, the direct interconversion between NAD and NADP represents another important strategy for regulating intracellular cofactor balance. The key enzyme facilitating this conversion is pyridine nucleotide transhydrogenase (PNT). Pyridine nucleotide transhydrogenase (PNT) catalyzes the reversible transfer of hydrogen ions between NADH and NADP^+^, enabling the interconversion of the two cofactor systems. PNT contains two isoenzymes, including membrane-bound PntAB and soluble UdhA [17]. PntAB catalyzes the energy-dependent transfer of reducing power from NADH to NADP^+^, while UdhA catalyzes the energy-independent reverse reaction (Figure 2). Since the activity of the PntAB hydrogen transferase requires no addition of external substrates, its expression is regarded as an effective means of regulating intracellular redox homeostasis. Heterologous overexpression of PntAB significantly enhances the synthesis of various cofactor-dependent metabolites. For example, overexpression of PntAB in *Bacillus licheniformis* could significantly increase bacitracin production, by enhancing NADPH regeneration capacity [18]. Additionally, several studies have demonstrated the feasibility and effectiveness of combining different strategies, which can maximize cofactor utilization and lead to more efficient product yields. Co-expression of NOX and PNT was shown to effectively mitigate ethanol stress of *Lactobacillus buchneri* by balancing intracellular NADPH levels [19].

These approaches optimize cofactor availability by redirecting metabolic flux, enhancing regeneration, and facilitating NAD/NADP interconversion to improve functional component biosynthesis in food-grade microorganisms [20]. However, their effectiveness may be limited by growth penalties and imprecision in targeting specific enzymatic reactions, highlighting the need for tailored interventions [21]. Integrating enzyme remodeling can enhance enzyme specificity and affinity, ensuring optimized cofactor pools are effectively utilized in targeted biosynthetic pathways [22].

## 3. Cofactor-Dependent Molecular Remodelling

Cofactor-dependent molecular *remodelling* is a precise engineering strategy for cofactors, designed to enhance catalytic efficiency and boost target product synthesis. This approach involves modifying enzyme structures or developing artificial cofactor *analogues* to reshape cofactor specificity and strengthen the affinity between enzymes and cofactors, thereby enabling a more effective metabolic match.

### 3.1. Alternation of Cofactor Preference Through Enzyme Modification

**Figure 3:**
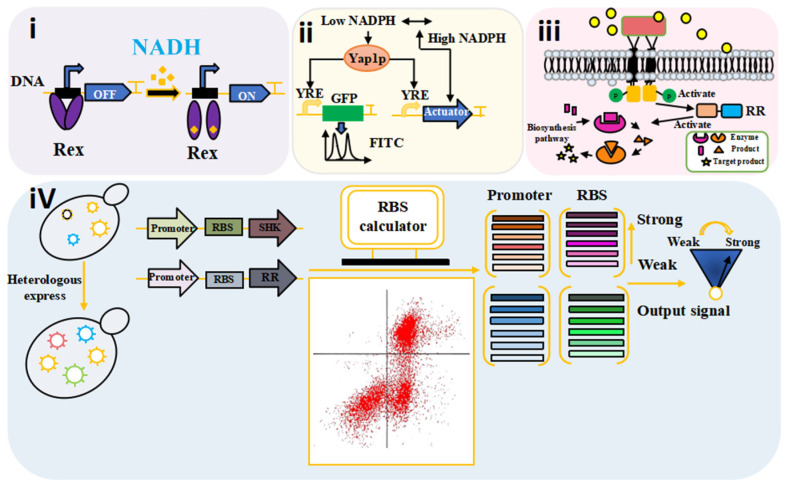
Application of NAD(H) and NADP(H) sensors and regulatory tools in living cells. (**i**) The redox-sensitive transcription factor Rex dynamically responds to the NADH/NAD^+^ ratio: when NADH levels are low, Rex binds DNA to repress ndh transcription; when NADH accumulates, Rex dissociates, allowing ndh expression and NAD^+^ regeneration. (**ii**) The Yap1p redox sensor detects NADPH/NADP+ balance and enables high-throughput screening of strains with optimized NADPH availability. (**iii**) The SoxR biosensor facilitates screening of NADPH-dependent enzyme mutants (alcohol dehydrogenase) for enhanced catalytic efficiency. (**iv**) The CSR-SALAD computational tool predicts cofactor specificity reversal mutations, enabling rational design of enzymes with tailored cofactor preferences. These tools collectively enable precise, real-time monitoring and engineering of cofactor homeostasis.

Many studies focus on the specific modification of enzyme cofactors by altering their affinity for different cofactors, through targeted mutations, insertions, or deletions in the amino acid sequences (Figure 3). The advancement of structural biology has elucidated the three-dimensional structures of many key enzymes that rely on cofactors. Notably, the protein binding mechanisms with NAD and NADP exhibit marked differences. The protein interactions with nicotinamide molecules are heavily influenced by substrate characteristics. NADP exhibits more flexibility in its interactions with proteins, while NAD complexes tend to be more conservative. Specifically, Aspartate and glutamate form hydrogen bonds with the adenine diol group of the NAD.NADP-specific binding proteins achieve selective recognition by forming a network of hydrogen bonds between the arginine side chain and phosphoester. Beyond the distinct conserved structural features of cofactor-binding enzymes, the specificity for NAD/NADP is largely influenced by the charge and polarity of the binding pocket [23].

Despite significant progress, existing technical bottlenecks still constrain applications in the food industry. These include enzyme activity loss caused by traditional mutagenesis methods, the impact of the conformation stability under food processing environments, and inefficiency caused by reliance on high-throughput screening. Additionally, computer simulations and machine learning algorithms can be utilized to more accurately predict the impact of amino acid mutations on cofactor binding. Meng et al. (2016) developed a novel computational protein design strategy to modify three key catalytic sites (D176S, I177R, F178T) of lactate dehydrogenase (LDH) in *Lactobacillus delbrueckii*, thereby creating a highly efficient bifunctional enzyme [24]. The mutant achieved a 184-fold enhancement in catalytic efficiency for NADPH, with a significant improvement for NADH. This dual-effect optimization arises from the synergistic enhancement of cofactor in both binding affinity and catalytic rate, demonstrating the flexibility of engineered enzymes in metabolic environments where NADH and NADPH coexist.

### 3.2. Utilization of Artificial Cofactor Analogues

The application of natural cofactors in food-grade enzyme engineering is limited by low solubility, poor environmental stability, and high costs. This has spurred interest in developing artificial cofactor systems for food biocatalysis. For example, malate dehydrogenase was engineered using directed evolution to modify its substrate-binding pocket, allowing it to utilize nicotinamide fluorocytidine dinucleotide (NFCD) instead of natural NAD^+^ for the conversion of L-malate. This marked the first successful application of efficient artificial cofactor regeneration in food-grade enzymatic reactions [25]. This breakthrough provided a methodological paradigm for the directed evolution of redox enzymes in food processing, while also opening orthogonal regulatory pathways in in vitro biosynthesis of functional food components. Novel artificial cofactors offer significant advantages in food-grade redox reactions, achieving higher catalytic conversion rates and enhanced stability under high-temperature and acidic conditions [26]. These create new opportunities for developing low-cost, robust food enzyme catalytic processes. However, in vivo applications encounter significant challenges, mainly due to the lack of compatibility between artificial cofactors and microbial endogenous metabolic networks. It is crucial to enhance their transmembrane transport efficiency and address competitive inhibition against natural cofactors.

Cofactor-dependent molecular remodeling complements cofactor pool regulation by addressing specificity bottlenecks, yet its static nature overlooks variability in food fermentation. This highlights the need for dynamic balance strategies for real-time adaptation and cofactor monitoring [27].

## 4. Dynamic Equilibrium of Cofactor Metabolic Networks

The dynamic equilibrium of cofactor metabolic networks represents a sophisticated strategy in food-grade microbial cofactor engineering. By leveraging synthetic biology, this approach constructs adaptive regulatory circuits based on redox-responsive transcription regulators and gene-encoded biosensors [3,28], enabling real-time monitoring and dynamic regulation of intracellular cofactor levels during fermentation, thereby enhancing the synthesis of target functional components [29,30].

### 4.1. Fine-Tuning of Redox-Related Transcription Regulators

Rex is a key redox-responsive transcription repressor, that plays a crucial role in maintaining the NADH/NAD+ balance in some Gram-positive bacteria [3]. It is a homodimer composed of an N-terminal DNA-binding domain and a C-terminal NADH-binding domain. Altering the C-terminal α-helix stabilizes the Rex homodimer, thereby inhibiting gene transcription [28]. Intracellular NAD+ concentrations are higher than those of NADH, promoting Rex binding upstream of NADH dehydrogenase (*ndh*) and repressing its transcription (Figure 3i). This repression leads to NADH accumulation. Conversely, when NADH concentrations exceed those of NAD+, Rex is released from its DNA binding site, activating *ndh* transcription and accelerating NADH oxidation to regenerate NAD+. This Rex-mediated feedback loop represents a dynamic equilibrium mechanism that enables real-time adaptation to fluctuating redox states. This dynamic equilibrium mechanism was applied to optimize probiotic fermentation processes. It significantly increased the production of functional metabolites including γ-aminobutyric acid (GABA) and extracellular polysaccharides, by stabilizing intracellular redox states [29]. The expression of Anr transcription activator was regulated by NADPH levels [30]. The Anr transcription factor cooperates with NADPH to regulate anthocyanin biosynthesis, thereby affecting the nutritional value of food [31]. HdfR is a transcription regulator that weakens NADPH-dependent glutamate synthesis [32].

### 4.2. Gene-Encoded Biosensors Precisely Regulate Cofactors

Synthetic biology provides an innovative technological platform for constructing efficient microbial cell factories in the food industry, by integrating non-natural biological components or natural modular components. Representative and powerful synthetic biology tools can promote the development of cofactor engineering. Gene-encoded biosensors serve as important tools for real-time monitoring of the intracellular metabolic status of food-grade microorganisms. As a key cofactor, the supply efficiency of NADPH directly affects the biosynthesis of terpenoids and essential amino acids. The SoxR biosensor could be used to screen for alcohol dehydrogenase mutants that mediated NADPH reactions in *Lactobacillus brevis* [33].

The Yap1p redox sensor established a screening strategy based on sensors/dose-sensitive genes (DSGs), to detect the NADPH/NADP+ balance (Figure 3ii). In metabolic engineering of *S. cerevisiae*, targeted regulation of the ALD6 gene significantly improved NADPH synthesis efficiency, surpassing the traditional ZWF1 pathway [34]. In addition, the Yap1p redox sensor could be used to identify gene knockout targets to improve NADPH cofactor homeostasis [35]. Traditional antibiotic screening mechanisms may disrupt cellular homeostasis during metabolic regulation, while the metabolic balance of microorganisms directly impacts product quality and safety in food fermentation. The Yap1p sensor/DSG screening technology could precisely detect endogenous signals, allowing for the enrichment of high-performance strains without antibiotics and avoiding the risk of antibiotic residues [36]. This strategy enables stable screening of large libraries in both liquid and solid media, demonstrating high-resolution advantages for the long-term evolution of microbial strains in the food industry (Figure 3iii), while reducing false-positive results caused by antibiotic degradation [37].

Cofactor Specificity Reversal-Structural Analysis and Library Automated Design (CSR-SALAD), a next-generation synthetic biology tool, provides an efficient solution for the directed evolution of food enzyme preparations (Figure 3iv). It takes specific enzyme information as input and uses automated structural analysis to predict potential mutations, thereby simplifying the process of reversing cofactor specificity [38]. This semi-rational, structure-guided strategy can modify many proteins efficiently, making the reversal of cofactor specificity more time-efficient and feasible. The CSR-SALAD-optimized alcohol dehydrogenase exhibited excellent specificity conversion efficiency in the NADPH regeneration system, significantly improving the bioconversion rate of D-tagatose [39], and was successfully applied to the modification of the food-related enzymes [40].

## 5. Discussion and Conclusions

NAD and NADP, play a crucial role in the microbes metabolic engineering of food-related microorganisms. They are essential for maintaining redox homeostasis and supporting the efficient biosynthesis of functional components. This review highlights innovative strategies for engineering these cofactors, including regulating the cofactor pool through metabolic flux optimization and regeneration systems, remodelling enzymes via targeted modifications and artificial analogues, and maintaining dynamic balance using biosensors and transcription regulators. These strategies effectively address redox imbalances during fermentation processes, with selected studies reporting yield increases ranging from approximately 30% to 180% for specific functional ingredients [9,24].

The demand for reducing power varies across different physiological stages of microbes, and traditional static methods have been shown to result in efficiency losses of up to 25% [34]. Consequently, dynamic regulation facilitated by biosensors and real-time cofactor monitoring is emerging as a crucial future direction for the application of synthetic biology in the food industry. Recent developments, such as the CSR-SALAD tool designed for enzyme specificity reversal, demonstrate the ability of computational metabolic models to predict and optimize cofactor dynamics [38], offering a promising approach for advancing food biomanufacturing. Despite these advancements, traditional metabolic engineering still faces significant challenges in achieving scalable and dynamic regulation of cofactor networks during fermentation. This highlights the urgent need for innovative, multidimensional approaches. Comprehensive studies of redox-driven carbon metabolism and the roles of global cofactor transcription regulators, such as Rex and Anr, are essential for identifying novel regulatory nodes. These investigations should leverage multi-omics technologies, including metabolomics and transcriptomics, to facilitate a thorough mapping of metabolic pathways and their interactions [41]. Additionally, developing advanced biosensing systems integrated with computational tools for dynamic cofactor balance control across cellular and industrial reactor scales is crucial [42]. For instance, optogenetic systems could enable the precise temporal regulation in solid-state fermentation processes, while predictive computational models could optimize flux distributions in complex mixed microbial systems, potentially reducing by-product formation as indicated by preliminary metabolic simulations. The integration of such synthetic biology tools into food-grade microbial platforms holds promise for scalable and precise control in industrial food biomanufacturing [43].

Addressing industrial challenges related to cost, safety and sustainability remains a critical priority in the field of cofactor engineering. While some of the strategies discussed rely on model organisms or cell-free systems, their translation to food-grade applications requires rigorous validation. Given that redox regulation, cofactor utilization, and transcriptional control are highly species- and condition-dependent, such validation must account for the specific metabolic context of the target microorganism. The genetic modification of strains intended for food use must adhere to stringent safety guidelines. For instance, regulatory frameworks established by the European Food Safety Authority (EFSA) and similar bodies mandate thorough safety assessments for genetically modified microorganisms used in food production. Although the adoption of artificial cofactors can potentially enhance production efficiency, it may also lead to increased costs compared to conventional methods. This underlines the urgent need for cost-effective alternatives, such as low-cost analogues or efficient recycling mechanisms [26]. It is equally important to implement rigorous safety evaluations for gene-edited strains to ensure consumer protection. Adhering to regulatory frameworks, including Food and Drug Administration guidelines, is essential for meeting food safety standards [22]. Insights from metagenomic mining studies suggest that this approach could facilitate the recovery of waste biomass as value-added food products, thereby contributing to industrial sustainability [44].

The key characteristics, advantages, limitations, and industrial readiness of three approaches to cofactor engineering provide a clearer framework for selecting appropriate strategies. Strategy I (cofactor pool regulation) is applicable to a wide range of food-grade microorganisms and is relatively simple to implement. However, static knockouts can impose metabolic burdens that reduce growth rates and are highly strain-dependent [45]. Strategy II (enzyme remodeling) allows for precise control over cofactor specificity but requires detailed structural knowledge and may lead to reduced enzyme stability during food processing. Strategy III (dynamic regulation) offers real-time adaptability, which is particularly valuable for large-scale fermentation processes where conditions fluctuate [46]. However, its implementation relies on sophisticated synthetic biology tools that may not be readily available in all industrial settings. A key trade-off exists between engineering complexity and industrial feasibility. While Strategies II and III provide greater precision and adaptability, they necessitate more extensive strain development and regulatory approval for food applications. In contrast, Strategy I, despite its static nature, remains the most readily implementable option for many current food fermentation processes.

These systematic innovations hold significant potential to advance the precise manufacturing of functional fermented foods, effectively tackling key industrial challenges while concurrently paving the way for sustainable resource utilization. Future research should focus on conducting pilot-scale validations of computationally driven dynamic systems to address the critical transition from laboratory achievements to industrial feasibility.

## Data Availability

No new data were created or analyzed in this study. Data sharing is not applicable to this article.

## Abbreviations

The following abbreviations are used in this manuscript:

NAD	Nicotinamide adenine dinucleotide
NADP	Nicotinamide adenine dinucleotide phosphate
TCA	Tricarboxylic acid cycle
PPP	Pentose phosphate pathway
LDH	Lactate dehydrogenase
PNT	Pyridine nucleotide transhydrogenase
PDH	Pyruvate dehydrogenase
EPS	Exopolysaccharides
FDH	Formate dehydrogenase
NOX	NADH oxidase
G6PD	Glucose-6-phosphate dehydrogenase
ME	Malic enzyme
GDH	Glutamate dehydrogenase
ADH	Alcohol dehydrogenase
NFCD	Nicotinamide fluorocytidine dinucleotide
Rex	Redox-sensing transcriptional repressor
ndh	NADH dehydrogenase
DSG	Dose-sensitive gene
CSR-SALAD	Cofactor Specificity Reversal-Structural Analysis and Library Automated Design
